# Clinical & Experimental Immunology: Highlights of 2021

**DOI:** 10.1093/cei/uxab031

**Published:** 2021-12-04

**Authors:** Leonie S Taams, Matthew Perryman

**Affiliations:** 1 Centre for Inflammation Biology and Cancer Immunology, Department of Inflammation Biology, King’s College London, London, UK; 2 British Society for Immunology, London, UK

Marked by a special birthday and multiple other events, 2021 has been another busy and exciting year for *Clinical & Experimental Immunology* (*CEI*). We pushed on with our publication plans focused around our restructured Editorial Board, reached our highest ever Impact Factor, and began looking ahead to new initiatives and developments. Here, we dive into some of our highlights from the past 12 months.

This year, *CEI* celebrated its 55th anniversary. We marked this significant milestone with a variety of activities across multiple platforms. We published an Editor-in-Chief interview [1], as well as an editorial [2] in the September issue of the journal, outlining some of *CEI*’s most significant achievements over the past 55 years. Additionally, our Section Editors shared their thoughts on some of the key developments in their fields since the journal’s launch [3].

Among our contributions to the immunology community were some state-of-the-art review series. In a follow-up to Part 1 of their ‘Immunology of Diabetes Society Review Series: Insights into Pathogenesis of Type 1 Diabetes’ series, Susan Wong, of Cardiff University, and Timothy Tree, of King’s College London, presented two articles around the topic [4]: Battaglia *et al*.’s insightful debate on which specific immune cells/targets are the ‘Achilles Heel of type 1 diabetes’ which was informed by similar discussions at the Immunology of Diabetes Society meeting in London 2018 [5], and Faridi *et al*.’s review which presented further insights into the generation of post-translationally modified peptides and their contribution to immune recognition [6]. In celebration of the 100th anniversary since the discovery of insulin, we also compiled a joint virtual issue with *Immunology & Cell Biology*, the flagship journal of the Australian and New Zealand Society for Immunology, that highlighted some of the key-related articles published in our journals [7]. For our second series, our final issue of the year saw the publication of the first of a two-part series titled ‘Imaging Immunological Processes in Neuroinflammatory Diseases’, led by Sandra Amor and David Owen. Part I presented six review articles exploring current approaches used to monitor disease and assess efficacy of treatment regimens in a range of neurodegenerative and neuroinflammatory diseases [8], with Part 2 of this series to follow in 2022.

Some of our favourite articles in this year’s *CEI* publications were highlighted as our Editors’ Choices. Richter *et al.* had one of the most well-received articles this year with their original research into the prevalence of autoantibodies in COVID patients [9]. Sippl *et al.* contributed another research article of note, reporting the possible involvement of synovial IL-17A and IL-6 in lupus arthritis [10], whilst Glennon-Alty *et al.* published research that identified complex regulatory signalling networks in which type I interferons profoundly alter the response of neutrophils to inflammatory cytokines [11]. The appointment of Dr Cindy Ma as our dedicated Section Editor for the Immunodeficiency Section has resulted in many excellent submissions in this area of research, some of which were selected as Editors’ Choices: del Pino Molina *et al.* demonstrated reduced expression levels of Bcl-2 in memory B cells from patients with common variable immunodeficiency (CVID), which may compromise the long-term survival of these cells [12]. Based on a pilot study conducted in Malaysia, Ripen *et al.* reported that whole-exome sequencing significantly enhances the capacity to diagnose inborn errors of immunity in CVID [13]. Rösler *et al.* reported a GM-CSF gain-of-function mutation in a family with Behçet’s disease-like disorder but without systemic inflammation [14]. Finally, Abdelwahab *et al.* found, in a study of Egyptian health care workers, that a specific TLR-9 genotype could predict the outcome of cell-mediated immune responses to hepatitis-C [15].

Our six Sections, covering Autoimmunity, Cancer Immunity, Immune-mediated Inflammatory Diseases, Immunodeficiency, Infectious Diseases and Vaccines, and Neuroimmunology, have been developing since we restructured our editorial processes in 2019. In 2021, we held our first virtual editorial board meetings with each section. These meetings allowed us to engage with our brilliant and supportive Editorial Board Members and benefit from their ideas and experience as authors, thereby placing us in good stead to continue evolving as a journal and capitalize on our progress to date. Our success so far was best summed up by our announcement earlier in the year of our highest ever Impact Factor of 4.330. Based on the citations in 2020 of our articles published the 2 years prior, it ranks us 78 out of 162 global immunology journals. We intend to build on this achievement and continue to take the journal from strength to strength.

From 2022, the BSI publishing portfolio will see some changes. *CEI*, the longest standing journal owned by the Society, will move to a new publisher, Oxford University Press (OUP). Here, *CEI* will join the BSI’s first Open Access journal, *Immunotherapy Advances*. We are very excited about this new partnership and have been working closely to ensure a seamless transition and impeccable author service during the move. The move will see *CEI* with a new look website, logo, and front cover designed to assimilate with those of *Immunotherapy Advances*. This and the new BSI–OUP partnership will allow the Society to build its family of journals and continue to support future generations of immunologists.

After this highly eventful year, the Chief Executive Officer of the BSI, Dr Doug Brown added: ‘2021 has marked a year of change within the BSI publishing portfolio, the launch of *Immunotherapy Advances*, saying goodbye to *Immunology*, and transitioning *CEI* to our new publishing partner. This could not have been done without the brilliant leadership of Leonie Taams, who is now entering her 6th year as Editor-in-Chief of the journal. With the support of her team of expert Section Editors, the journal will continue to go from strength to strength. We are confident that we have secured a bright future for all our journals that allows them to flourish with a partner who understands and supports the goals of the BSI and *CEI*’.
